# Supplementary material for: The compressive strength of crumpled matter
Source: Nat Commun. 2019 Apr 3;10:1502. doi: 10.1038/s41467-019-09546-7 (PMC6447532; doi:10.1038/s41467-019-09546-7)
Supplement: Supplementary file 1 — Supplementary Information [file 41467_2019_9546_MOESM1_ESM.pdf]

Supplement to Compressive Strength of Crumpled Matter

Andrew B. Croll*

Department of Physics and Materials and Nanotechnology Program,

North Dakota State University, 58102 Fargo, USA.

Timothy Twohig

Department of Physics, North Dakota State University, 58102 Fargo, USA.

Theresa Elder

Materials and Nanotechnology Program,

North Dakota State University, 58102 Fargo, USA.

(Dated: March 12, 2019)

*Electronic address: andrew.croll@ndsu.edu

SUPPLEMENTARY DISCUSSION

Föppl-von Kármán Number: It is not uncommon to classify mechanical experiments with thin films by their range of Föppl-von Kármán numbers (FvK), defined as $\gamma = L^2/h^2$, where L is the lateral size of a film and h its thickness. For example, scaling behaviour is often limited to a certain range of FvK numbers. In the present work, we characterized the force required to compress a crumpled film by an amplitude (F_0) and a scaling exponent (α) related through the power law: $F = F_0 x^\alpha$, with F the applied force and x the gap size. Given the variation we observed in both quantities, it was natural to check for correlation with the FvK number to see if the variation of our experiments was simply due to our approach to an asymptotic limit.

Supplementary Figure 1 shows a clear lack of correlation between power law amplitude (a.) or exponent (b.) and the Föppl-von Kármán number. For example, as the FvK number increases we see no signs of the measurements approaching a constant value. We note our experiments are limited to only about 8 orders of magnitude in FvK number, which could (in principle) hide any trends occurring at higher FvK numbers.

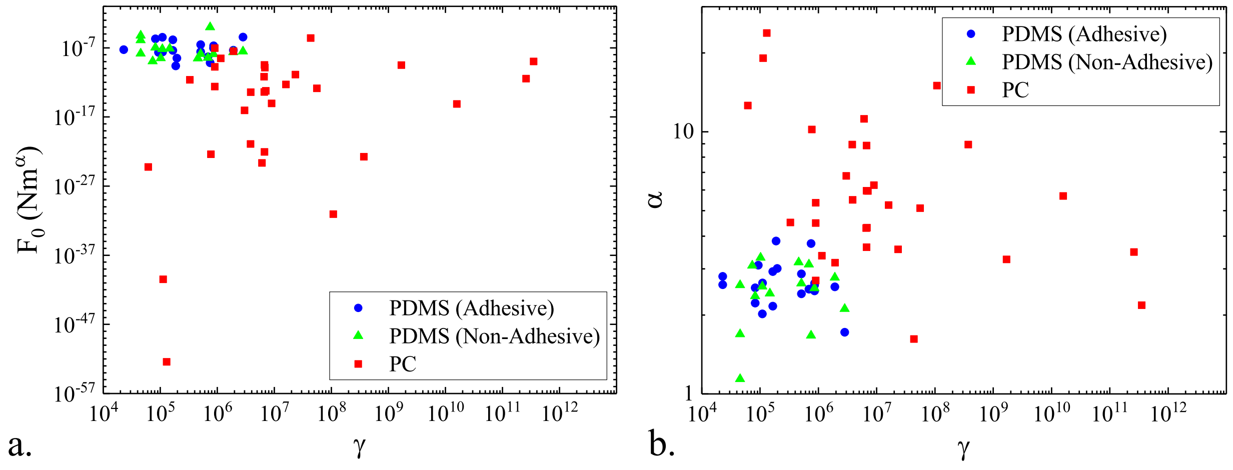

Supplementary Figure 1. Relation between the Föppl-von Kármán number, γ, and the power law describing the compression of a crumpled film. **a** amplitude versus FvK number. **b** scaling exponent versus FvK number. Data appears uncorrelated in the range available to experiments.

Ridge Model: Matan and coworkers proposed a scaling model to describe the compaction of a crumpled film between two walls[S2]. The model considered the stretching in-

curred in a ridge joining two developable cones as the primary energetic cost in the system, and estimated the number and size of ridges from the density of the crumpled film. Specifically, the model predicts compaction force to scale as:

$$F \sim Eh^{8/3}L^{16/3}R^{-10/3}H^{-8/3}, \tag{S1}$$

where E is Young's Modulus, h is film thickness, L is the lateral extent of the film, R is the crumple's radius, and H is the gap between the compressing walls. As noted in the manuscript, we find exponents similar to 8/3 only in the PDMS films suggesting the argument is plausible for elastic materials. However, it is important to check the scaling of the force with film dimensions and crumple radius as these are also prominent features of the model. If all force-compression data is fit with a power law ($F = F_0 x^{\alpha}$) then the remaining variables can examined in a plot of F_0 as a function of h, L and R.

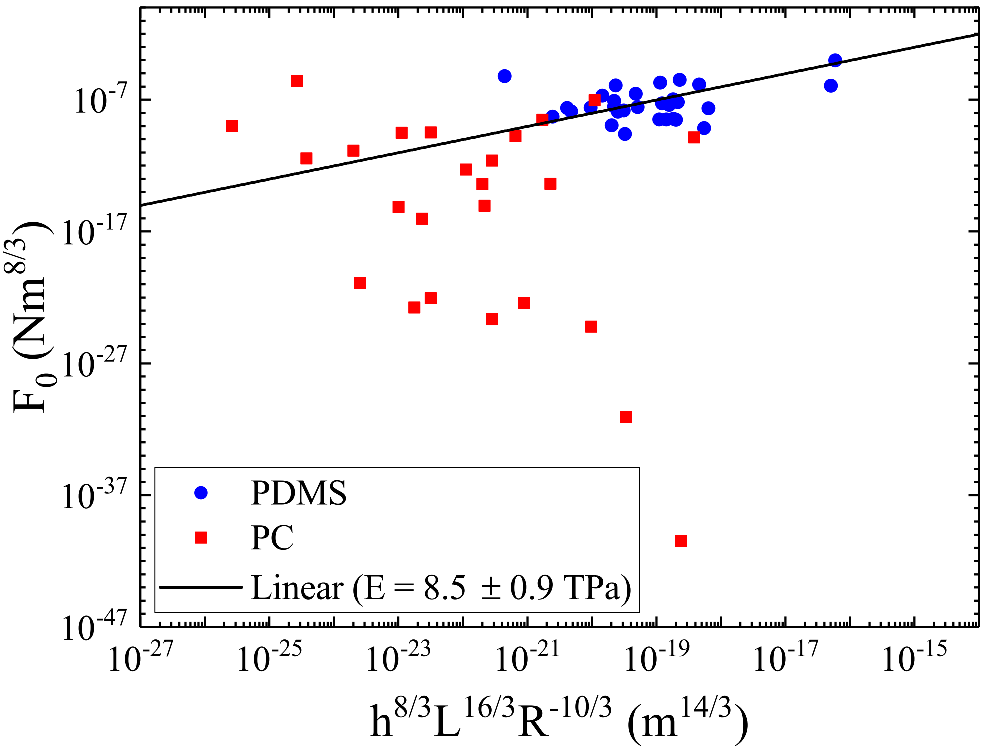

Supplementary Figure 2. Scaling prediction of the Matan et al. model. The large scatter in the data makes it difficult to confirm the linear fit with either material, though it is plausible for the PDMS data. Quantitatively, the PDMS data predicts a modulus of 8.5 TPa, which is many orders of magnitude from the known modulus of 2 MPa. Such disagreement is beyond any reasonable factors neglected in Eqn. S1

Supplementary Figure 2 shows the force amplitude of both PDMS and PC data. The PC data shows considerable scatter, and makes it unlikely that a linear fit is reasonable. This is consistent with the high, and variable, scaling exponent measured with respect to the confining dimension. Both observations are clear signs that PC is not well described by Eqn. S1. The PDMS data of Supplementary Figure 2 shows scatter, but is plausibly consistent with a linear trend (shown as a solid line). On this axis, the slope of the liner fit is interpreted as the film's modulus and for the PDMS shown yields a modulus of 8.5 ± 0.9 TPa, far beyond what is reasonable for PDMS. Once again it must be concluded that Eqn. S1 is not, in fact, consistent with the data.

Fold Model: Deboeuf et al. proposed an alternative model based on the dominance of the energetic cost of single, fully collapsed ridges (e.g. curvature $\rightarrow 1/h$) in crumpling a film[S2]. The model predicts compressive force to scale as $F \sim Eh^2(x/L)^{-\alpha}$, where alpha depends on the exact type of folding but ranges from 1 to 4. Supplementary Figure 3 shows F_0 as measured for PDMS and PC as a function of $h^2 L^\alpha$. A linear fit to the PC data is also shown in the figure. As in Supplementary Figure 2, consistency with a linear correlation means the predicted scaling is accurate and the slope is the modulus up to a scaling constant. However, we observe the correlation to be imperfect and the modulus to be inaccurate by several orders of magnitude (we find a modulus of 7 MPa for PC). The PDMS data ranges only a few orders of magnitude but has a similar error in correlation. More importantly, 3D imaging shows very few fully collapsed folds in either material - counter to the foundational assumption of the model. PDMS, in fact, rarely showed any sizable regions of curvature $\rightarrow 1/h$. Additionally, the reader is reminded of the high scaling exponent observed for PC which is also inconsistent with the predictions of the fold model. We conclude that the scaling predicted by the fold model appears close to what is found in experiment, but the model itself is not fully consistent with observations.

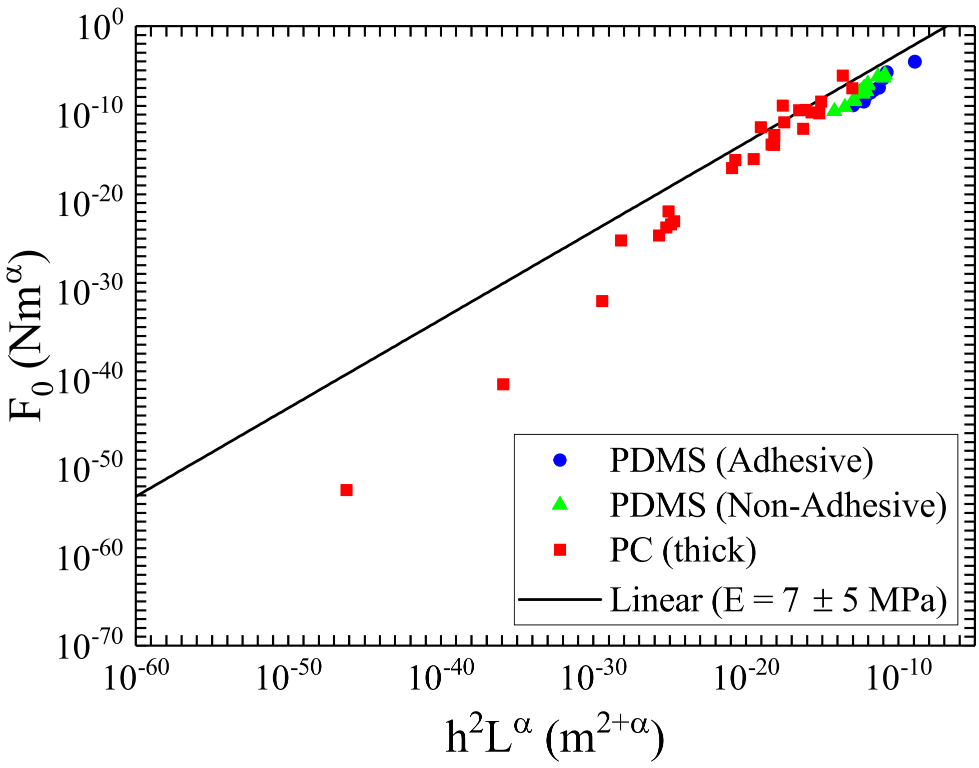

Supplementary Figure 3. Scaling predicted by fold model. A linear fit to the PC data is shown as the solid line, resulting in a modulus of 7 MPa, far below what is expected.

SUPPLEMENTARY REFERENCES

S1. Matan, K., Williams, R., Witten, T. & Nagel, S. Crumpling a thin sheet. Phys. Rev. Lett. **88**, 076101 (2002).

S2. Deboeuf, S., Katzav, E., Boudaoud, A., Bonn, D. & Adda-Bedia, M. Comparitive study of crumpling and folding of thin sheets. Phys. Rev. Lett. **110**, 104301 (2013).
